# Differentiating solitary brain metastases from high-grade gliomas with MR: comparing qualitative versus quantitative diagnostic strategies

**DOI:** 10.1007/s11547-022-01516-2

**Published:** 2022-06-28

**Authors:** Ioan Paul Voicu, Emanuele Pravatà, Valentina Panara, Riccardo Navarra, Peter A. Mattei, Massimo Caulo

**Affiliations:** 1 Department of Imaging, “G. Mazzini” Hospital, 64100 Teramo, Italy; 2 Neurocenter of Southern Switzerland, Neuroradiology Department, Ospedale Regionale di Lugano, via Tesserete 46, 6901 Lugano, Switzerland; 3 Department of Neuroscience and Imaging, ITAB-Institute of Advanced Biomedical Technologies, University G. d’Annunzio, Chieti, Italy; 4 Department of Radiology, University “G. d’Annunzio” of Chieti, Chieti, Italy

**Keywords:** Brain neoplasms, Magnetic resonance imaging, Glioma, Neoplasm metastasis, Perfusion, Area under curve

## Abstract

**Purpose:**

To investigate the diagnostic efficacy of MRI diagnostic algorithms with an ascending automatization, in distinguishing between high-grade glioma (HGG) and solitary brain metastases (SBM).

**Methods:**

36 patients with histologically proven HGG (*n* = 18) or SBM (*n* = 18), matched by size and location were enrolled from a database containing 655 patients. Four different diagnostic algorithms were performed serially to mimic the clinical setting where a radiologist would typically seek out further findings to reach a decision: pure qualitative, analytic qualitative (based on standardized evaluation of tumor features), semi-quantitative (based on perfusion and diffusion cutoffs included in the literature) and a quantitative data-driven algorithm of the perfusion and diffusion parameters. The diagnostic yields of the four algorithms were tested with ROC analysis and Kendall coefficient of concordance.

**Results:**

Qualitative algorithm yielded sensitivity of 72.2%, specificity of 78.8%, and AUC of 0.75. Analytic qualitative algorithm distinguished HGG from SBM with a sensitivity of 100%, specificity of 77.7%, and an AUC of 0.889. The semi-quantitative algorithm yielded sensitivity of 94.4%, specificity of 83.3%, and AUC = 0.889. The data-driven algorithm yielded sensitivity = 94.4%, specificity = 100%, and AUC = 0.948. The concordance analysis between the four algorithms and the histologic findings showed moderate concordance for the first algorithm, (*k* = 0.501, *P* < 0.01), good concordance for the second (*k* = 0.798, *P* < 0.01), and third (*k* = 0.783, *P* < 0.01), and excellent concordance for fourth (*k* = 0.901, *p* < 0.0001).

**Conclusion:**

When differentiating HGG from SBM, an analytical qualitative algorithm outperformed qualitative algorithm, and obtained similar results compared to the semi-quantitative approach. However, the use of data-driven quantitative algorithm yielded an excellent differentiation.

## Introduction

High-grade gliomas (HGG) and cerebral metastases are the most common tumors of the brain in the adult population [1]. Differentiating a HGG, an aggressive tumor with a poor prognosis, from a solitary brain metastasis (SBM) has important prognostic and therapeutic implications. This can be achieved through numerous methods ranging from conventional reporting to multiparametric data-driven approaches.

Current treatment of HGG is based on surgery followed by the Stupp protocol [2, 3]. Cerebral metastases can present as a single or as multiple enhancing lesions. In less than 30% of patients with primary systemic tumor, cerebral mass is the first clinical manifestation. When a primary systemic tumor is suspected, comprehensive whole-body staging should be performed before making further therapeutic decisions [4]. Current treatment options for a SBM include surgery or Gamma-knife surgery and whole brain radiation therapy [5].

Several previous studies reported that differentiating an SBM from HGG with MRI alone can be difficult [6–11] since the two lesions may present with similar morphological characteristics. Several algorithms have been proposed for differentiating between the two lesions with conventional and advanced MRI techniques, including automated/semi-automated methods protocols [6–11]. These studies highlighted the importance of the peritumoral region; i.e., the high signal in T2-sequences surrounding the enhancing lesion. They found that the peritumoral region was purely vasogenic edema in metastases, but presented infiltration of neoplastic cells in HGGs [1] determining an increase in CBV values on perfusion MR. Perfusion-based methods were able to distinguish HGG from metastases with a pooled sensitivity and specificity of 90% (95% CI, 84–94%) and 91% (95% CI, 84–95%), respectively [12].

It was previously shown that the employment of a data-driven algorithm using multiparametric MRI may simplify this task and improve diagnostic accuracy in differentiating glioma grades over both the semi-quantitative and qualitative algorithms [13]. Therefore, we hypothesized that within the spectrum of options for differentiating HGG from SBM, a data-driven approach would yield superior diagnostic accuracy to the radiologist, compared to qualitative, standardized qualitative, and semi-quantitative diagnostic algorithms.

## Materials and methods

This retrospective study was approved by the Ethics Committee of the University “G. d'Annunzio” of Chieti-Pescara, Chieti, Italy, and complied with the Declaration of Helsinki. Written informed consent was obtained from all patients prior to their undergoing MR imaging in an ongoing study for brain tumor characterization and presurgical planning.

All patients with a single-enhancing brain lesion with the longest axis between 10 and 35 mm identified in a database containing 252 patients with HGG and 403 patients with brain metastases who had undergone MR imaging from January 2011 to January 2019. Exclusion criteria were butterfly lesions crossing the midline [14], and previous surgery, chemotherapy, and radiation therapy. Neither little or no peri-enhancing edema (high T2 signal alteration surrounding the enhancing area of the tumor) nor the presence of hemorrhage were considered exclusion criteria.

Patients underwent a standardized multimodal imaging protocol that included conventional and advanced MR sequences: DWI-ADC sequences and DSC T2* perfusion sequences (T2*PWI). Images were acquired with a 3 T MR imaging system (Philips Achieva X Series; Philips Medical System, Best, the Netherlands) using a sensitivity-encoding eight-channel head coil. Conventional and advanced MR imaging sequence parameters are provided in Table 1. Raw data were transferred to a PC workstation (Extended MR Workspace, release 2.6.3.2; Philips Medical Systems) for post-processing.

**Table 1:** MR-imaging parameters employed for data acquisition

Sequence	Parameters
Pre- and post-gadolinium enhanced	0.1 ml/kg gadobutrol (Gadovist; Bayer Schering Pharma, Berlin, Germany) administered
Three-dimensional turbo- field-echo T1-weighted	Sagittal acquisition; repetition time (ms)/echo time (ms), 7.6/3.7; slice thickness, 1 mm; matrix, 256 × 256
Turbo spin-echo T2-weighted	3-mm axial and coronal acquisition; 3000/80; matrix, 300 × 256
Fluid-attenuated inversion recovery	3-mm axial acquisition; 11,000/125; inversion time (ms), 2800; matrix, 320 × 200
Diffusion-weighted imaging	Single-shot echo-planar imaging; 28 Sects. (4 mm) obtained (3700/67; matrix, 128 × 128; b values, 0–1000 mm^2^/s)
Perfusion-weighted imaging	Intravenous injection of 2 ml of contrast medium at a flow rate of 2 ml/sec, followed by a 20-ml saline flush, was performed prior to perfusion-weighted imaging to minimize underestimation of CBV owing to the T1-shortening effect from potential contrast medium extravasation; after 3 min, dynamic T2*-weighted fast field-echo echo-planar imaging was performed (1576/40; 25 sections [4 mm] and matrix of 96 × 96); a series of 50 such volumes was acquired during an intravenous bolus injection of 0.1 mmol per kilogram of body weight of contrast media at a flow rate of 4 ml/sec, followed by a 20-ml saline flush

Each patient was evaluated with four different algorithms in a serial fashion to mimic the clinical setting where a radiologist would typically seek out further findings to reach a decision. This was performed independently by two radiologists (V.P. and E.P., with 7 and 10 years of experience, respectively) blinded to the histologic evaluation of the lesions. Disagreements were resolved by both readers in consensus with a third senior radiologist (M.C., 20 years of experience).

**Fig. 1:**
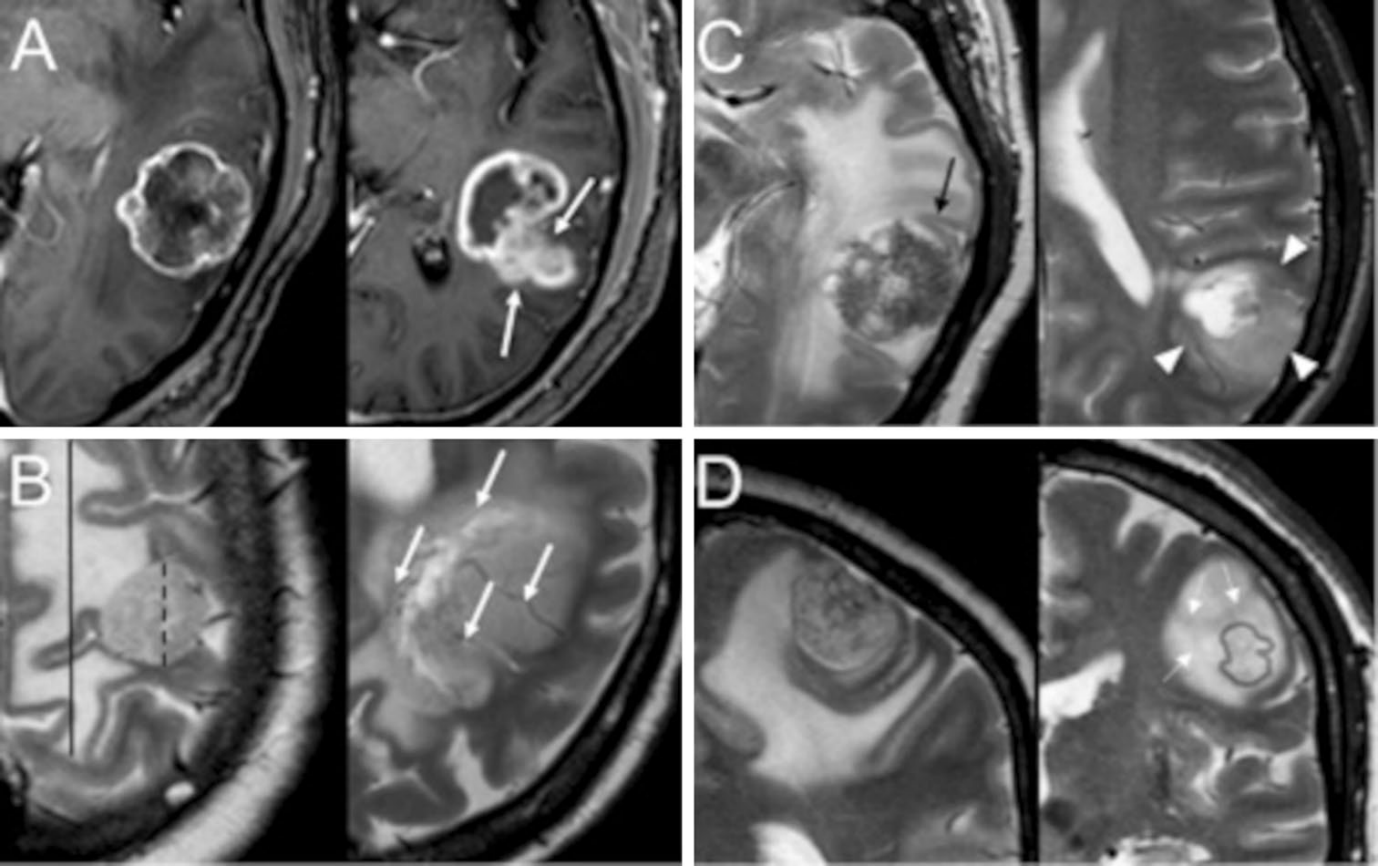
Analytic qualitative criteria used to differentiate single metastases (MET) from high-grade gliomas (GBM). **a** morphology and margins characteristics on post-contrast T1-weighted (T1w) axial images of a 66-year-old man with SBMMET from colon cancer (left), and in a 65-year-old man with GBM (right). SBM has an almost spherical shape and well-defined margins (arrows). In contrast, GBM exhibits an irregular shape, and areas with poorly defined margins. **b** Edema/lesion ratio and macroscopic vascularization on T2w axial images in a 56-year-old woman with SBM from breast cancer (left) and in a 71-year-old man with GBM (right). SBM has a high ratio between edema (continuous caliper) and lesion (dashed caliper). No prominent vessels coursing within the lesion are seen. In contrast, GBM has a relatively lower edema/lesion ratio and prominent intralesional vessels (arrows). **c** Lesion relationship with the cortex on T2w axial images, in a 66-year-old man with SBM (left, same as A) and in a 62-year-old man with GBM (right). SBM shows no thickening or definite signal change of the cortex in proximity of the lesion (arrow). However, GBM causes thickening and blurring of the cortex interface, suggesting infiltration (arrowheads). **d** T2-signal texture characteristics in the peri-enhancing region: coronal images of a 67-year-old woman with SBM from breast cancer (left) and a 75-year-old woman with GBM (right). Light gray outlines mark the corresponding enhancing nodules as seen on post-contrast T1w images. Whereas SBM exhibits a uniformly bright signal in the peri-enhancing region, suggesting simple vasogenic edema, GBM shows adjacent white matter signal inhomogeneity, with subtle hypointensities (arrowheads) suggesting tumor infiltration

*Qualitative algorithm.* Each reader provided an opinion regarding the expansive (more indicative of a metastasis) or infiltrative (more indicative of a glioma) nature of the tumor based on visual inspection of conventional imaging alone [15].
2. *Analytic qualitative algorithm*. The differentiation of the lesions was based on location and morphological features reported in the literature. The parameters were as follows: (a) supratentorial or infratentorial location of the lesion [16], (b) maximum diameter of the enhancing lesion and (c) peri-enhancing edema on an axial plane, (d) form and (e) margins of the enhancing lesion, (f) contrast enhancing of the cortex, (g) abnormal signal and/or thickening of the cortex not involved by the enhancing lesion [17–20], (h) low T2 signal regions in the peri-enhancing region[21], and (i) prominent blood vessels passing through the lesion [22]. All analytic qualitative parameters are summarized in Fig. 1 and Table 2.

**Table 2:** Parameters employed for the analytic qualitative approach (Algorithm 2)

Site	Supratentorial or infratentorial
Sequence	Parameters
T1-weighted post-contrast	Axial dimensions of the enhancing lesion (in mm)
	Shape of the enhancing lesion (regular, partly irregular, irregular)
	Margins of the enhancing lesion (regular, partly irregular, irregular)
	The enhancing lesion involves extensively the cortex?
T2-weighted on various planes	Axial dimensions of the high signal region surrounding the enhancing lesion (mm)
	Margins of the signal alteration corresponding to the enhancing lesion (regular, partly irregular, irregular)
	Are there prominent blood vessels passing through the lesion?
	Is there any abnormal signal and/or thickening of the cortex outside of the area corresponding to the enhancing region?
	Are there low T2 signal alterations in the peri-enhancing region?
FLAIR	Is there any abnormal signal and/or thickening of the cortex outside of the area corresponding to the enhancing region?
*Semi-quantitative algorithm*: Five regions of interest (ROIs) smaller than 1 cm.^2^ were positioned in three different regions: (a) one ROI in the contrast-enhancing regions, (b) three ROIs in regions with or without high signal intensity on T2-weighted images selected in the peri-enhancing region within 1 cm from the contrast-enhancing lesion, and (c) one ROI in areas of normal-appearing white matter in the contralateral hemisphere (Fig. 2). They were positioned to avoid partial-volume errors from adjacent non-tumor tissue and between enhancing and non-enhancing regions. Blood volume maps were generated from perfusion-weighted imaging data, from which nrCBV (normalized relative cerebral blood volume [CBV] divided by CBV in the normal-appearing contralateral white matter) [23]. Curve maximum height and curve recovery percentage of blood volume maps were obtained in each area. ADC maps were generated from diffusion-weighted imaging data and ADC values were obtained in each area. The PWI and ADC metrics in the peritumoral edema were calculated. The radiologists were then asked to provide an opinion on the nature of the lesions by supplementing morphological information with PWI and ADC values based on the reported cutoffs in the literature [1, 11]

**Fig. 2:**
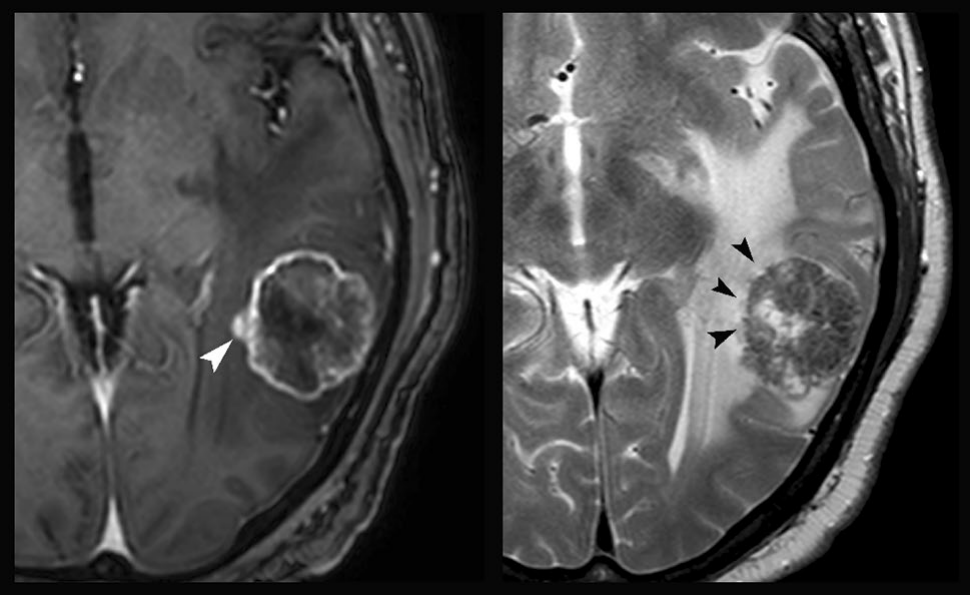
ROI positioning on the images of a patient affected by SBM (same patient as in images a and c from Fig. 1): post-contrast T1-weighted (left) and axial T2-weighted (right) images. The white arrowhead indicates the ROI positioned in the enhancing region. The three black arrowheads indicate the ROIs positioned in the peri-enhancing lesion within 1 cm from the enhancing lesion. A fifth ROI (not shown) was positioned in the white matter of the contralateral hemisphere for normalization purposes

Data-driven quantitative algorithm: The same parameters extracted in Algorithm 3 were used in the data-driven analysis algorithm reported below.

### Statistical analysis

Descriptive statistics for demographic characteristics and MRI parameters were reported using mean and standard deviation or median and interquartile range, as appropriate.

*Quantitative analysis algorithm:* A two-tailed independent sample *t* test was used to evaluate differences for each parameter and/or area utilized in the previous evaluations between HGG and SBM with an arbitrary cutoff of *p* < *0.*05, selected to reduce the number of variables that would be inserted in the data-driven model while avoiding an excessive risk of type II error [13].

Numerical cutoffs of the variables that survived this first test were determined with receiver operating characteristic (ROC) curve analysis. Variables that yielded statistically significant results in this second analysis (*p* < *0.*05) were then used in a discriminant function analysis (DFA), yielding standardized coefficients that indicate the unique contribution of each predictor variable to the function [13]. The model thus produced quantified the contribution of each individual variable in discriminating between the tumor types and determined whether the combination of these variables (an index identified as the “GM index”) was a predictive model for group membership. The model was verified by using the leave-one-out analysis.

*Diagnostic accuracy assessment:* Kendall coefficient of concordance test was performed to assess the agreement between each of the four types of evaluation (qualitative, analytic qualitative, semi-quantitative, and data-driven) and the reference standard (histologic examination). The *k* value was interpreted as fair (*k* ≤ 0.4), moderate (0.4 < *k* ≤ 0.6), good (0.6 < *k* ≤ 0.9) and excellent (*k* > 0.9). For each of the four algorithms, ROC analysis was then used to determine the optimal cutoff with relative sensitivity, specificity, and accuracy.

## Results

Thirty-six patients (10 women; mean age 65.5 years ± 10.6, range: 44–86 years) with a single enhancing brain lesion and a histologically proven diagnosis of previously untreated HGG (*n* = 18) or SBM (*n* = 18) were enrolled. The primitive tumors of the latter group were lung (*n* = 6), gastrointestinal tract (*n* = 5), breast (*n* = 3), melanoma (*n* = 3) and kidney (*n* = 1). The mean ages for patients with high-grade gliomas and single metastases were 64.06 ± 12.03 (range, 44–86 years), and 67.0 ± 9.2 (range, 52–82 years), respectively.

**Fig. 3:**
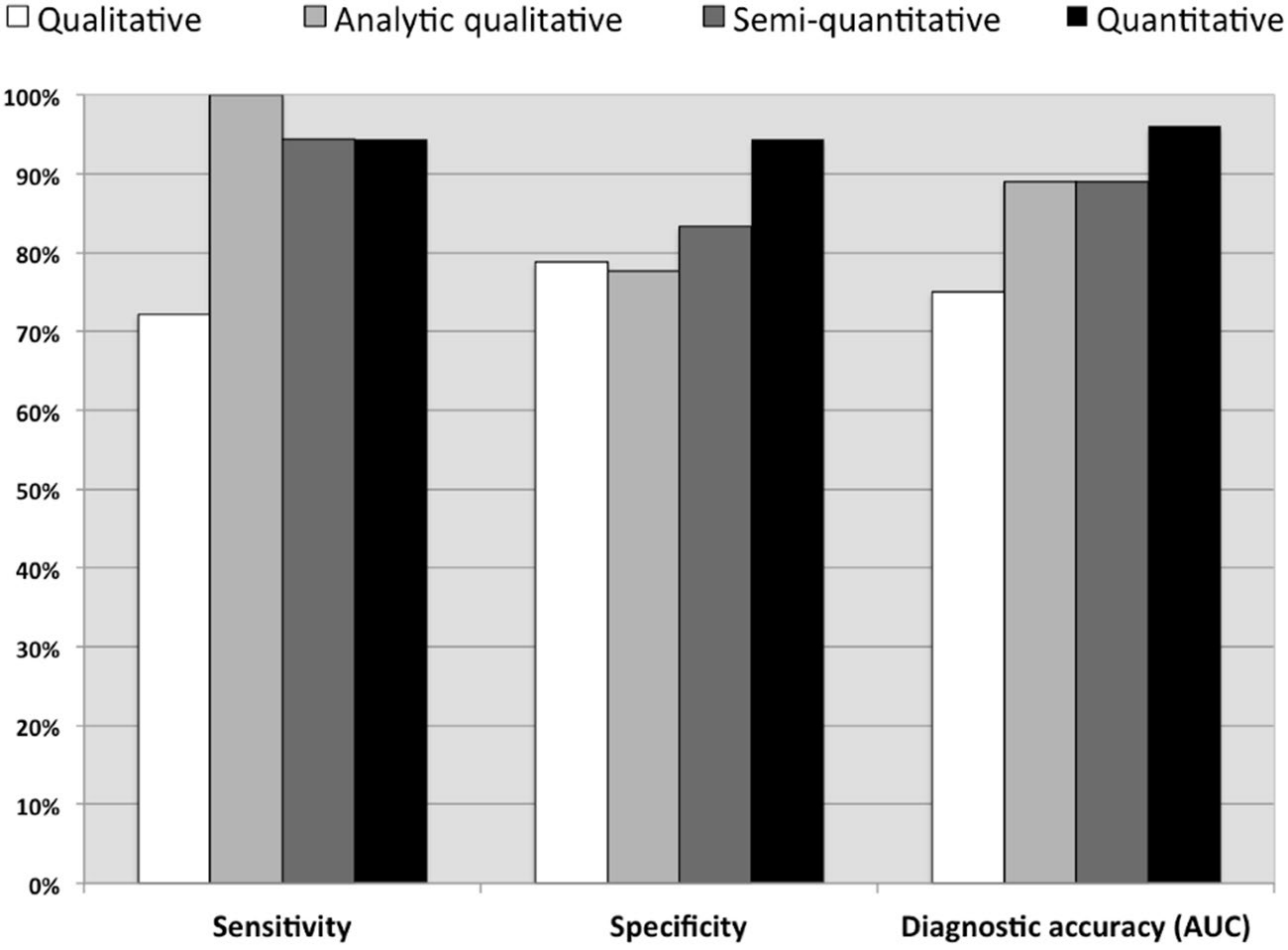
Bar graph shows the comparison of sensitivity, specificity and AUC values in distinguishing between high-grade gliomas and single brain metastases for the four diagnostic algorithms

Algorithm 1 (qualitative analysis) distinguished high-grade gliomas from metastases with a sensitivity of 72.2%, a specificity of 78.8%, accuracy of 75% and AUC of 0.75. Algorithm 2 (analytic qualitative analysis) distinguished HGG from SBM with a sensitivity of 100%, specificity of 77.7%, accuracy of 88.9% and an AUC of 0.889 (*p* < 0.01, Fig. 3). Algorithm 3 (semi-quantitative analysis), applying a-priori cutoffs reported in the literature, failed to substantially improve overall sensitivity and specificity over the analytic qualitative approach (sensitivity = 94.4%, specificity = 83.3%, accuracy of 88.9%. AUC = 0.889; *p* < 0.001).

As for Algorithm 4, the parameters that presented the highest significant difference between HGG and SBM are reported in Table 3 and the leave-one-out analysis did not yield modify the classification of any patients. These parameters (Mean T2nrCBV, Mean T2PRC, T2aPRC and T2cPRC) were evaluated by using a DFA to predict whether the lesions were HGG or SBM. This yielded a significant role for all predictors (*p* < 0.01). The results of ROC analysis of the GM index obtained with the data-driven analysis yielded a cutoff value of 0.321 for the GM index for distinguishing HGG from SBM, with a sensitivity of 94.4%, specificity of 94.4%, accuracy of 94.4% and AUC of 0.96 (*p* < 0.01, Fig. 3, Table 3).

**Table 3:** Parameters resulting from the discriminant function analysis (DFA) to discriminate between HGG and SBM

Parameters	Coefficients
Quantitative parameters	
Mean T2nrCBV	0.082
Mean T2PRC	−0.110
T2aPRC	0.113
T2cPRC	0.082
Constant value	−6.989

Table 4 reports diagnostic performance of each of the four Algorithms.

**Table 4:** Results. Sensitivity, specificity, accuracy and area under the curve (AUC) values are reported for each of the four analysis approaches employed to discriminate solitary metastases from HGG

Algorithm		Sensitivity (%)	Specificity (%)	Accuracy (%)	AUC
1	Qualitative analysis	72.2	78.8	75	0.75
2	Analytic qualitative analysis	100	77.7	88.9	0.89
3	Semi-quantitative analysis	94.4	83.3	88.9	0.89
4	Quantitative analysis	94.4	94.4	94.4	0.96

Among the quantitative parameters, those that could discriminate between the two lesions were the mean rCBV and meanT2PRC, respectively, with cutoffs of 1.46 (sensitivity 83%, specificity 89%, AUC 0.904, *p* < 0.01), and of 87.5% (sensitivity 88.9%, specificity 94.4%, AUC 0.907, *p* < 0.01).

The concordance analysis between the four algorithms and the histologic findings yielded significant moderate concordance for Algorithm 1, (*k* = 0.501, *p* < 0.01), good concordance for both Algorithm 2 (*k* = 0.798, *p* < 0.01), and Algorithm 3 (*k* = 0.783, *p* < 0.01), and excellent concordance for Algorithm 4 (*k* = 0.901, *p* < 0.0001).

## Discussion

Correctly diagnosing SBM or HGG is fundamental for therapeutic planning because treatment strategies vary significantly between these two types of lesions [2, 4, 5]. Several studies addressed the topic employing both conventional and advanced MRI techniques [1, 4, 6–12, 16–20, 24, 25]. This study evaluated the diagnostic performance of four different diagnostic strategies with an ascending order of parameter analysis and standardization: pure qualitative, analytic qualitative, semi-quantitative and data-driven. The data-driven algorithm with the GM index calculation yielded an excellent diagnostic performance (sensitivity of 94.4%, specificity of 94.4%, accuracy of 94.4% and AUC of 0.96) using a cutoff value of 0.321. A lower rank diagnostic performance (good) was achieved by both semi-quantitative and analytic qualitative algorithms, whereas the qualitative algorithm exhibited the worst performance.

### *Qualitative* algorithm

In our sample population, the qualitative algorithm, in which the neuroradiologists had to give an opinion based on the expansive or infiltrative aspect of the tumor alone, yielded comparable results with other qualitative evaluations reported in the literature, thereby exhibiting a relatively poor performance [16–18]. This is in line with previous studies, where lack of a clear radiological distinction. especially for gliomas, between expansive versus infiltrative features were found [16, 26, 27].

### *Analytic qualitative* algorithm

The analytic qualitative algorithm, exploiting a more standardized evaluation of several morphologic lesion features, achieved a better diagnostic performance. Two factors may contribute to explain the diagnostic improvement. First, combining several parameters could improve the diagnostic yield, as opposed to a single parameter evaluation (expansive/infiltrative pattern) of the lesion. Second, it is possible that technological improvements and routine use of high field MRI (that include improved T2 signal-to-noise ratio, higher spatial resolution, and thinner slice-thickness) could show morphological aspects of the lesions that could not have been previously evaluated in such detail. These results support the central role of the experienced radiologist in accurately discriminating between the two lesions.

### *Semi-quantitative *algorithm

The semi-quantitative algorithm, combining the morphologic evaluation with diffusion and perfusion parameter cutoffs taken from the literature, was comparable to the performance of the analytic qualitative algorithm, but achieved a better ranking than the qualitative algorithm. With respect to several previous studies [1, 4, 6–11, 24, 25], which reported that advanced techniques may improve diagnostic accuracy when compared to conventional imaging alone, the present results empathize the benefits of a structured and systematic algorithm of conventional imaging. In fact, the analytic qualitative algorithm employed in this study led to a superior performance than the unstandardized assessment of conventional imaging. Therefore, we argue that an analytical and systematic algorithm to reporting based on morphological characteristics seen on conventional imaging may still play an important role in brain tumor characterization.

These results do not go against the use of advanced MR techniques (especially perfusion) since these techniques remain fundamental for differentiating tumor and tumor-like lesions and for glioma grading. The results of this study support the relevance to the role of the neuroradiologist, who through a systematic reading of MR images can distinguish between HGG and SBM with a very good diagnostic accuracy.

### Data-driven analysis

The data-driven multiparametric algorithm yielded the highest diagnostic accuracy. We assert that this data-driven approach could present some potential advantages and may be implemented in a clinical reading setting, being inexpensive and requiring relatively little time (on the order of a few minutes) to process the results once the relevant data was extracted. The data-driven algorithm should not be intended as a replacement to the radiologist, but rather as a clinical tool to aid radiologists in discriminating between the two lesions.

Although multiparametric studies employing MR spectroscopy [1, 4–6] and diffusion tensor metrics [7] reported good or excellent results in differentiating HGG from SBM, our approach included the least time-consuming sequences T2*PWI and ADC, requiring an acquisition time and data analysis process of less than 5 min. On the other hand, other studies based on advanced metrics employed semi-automated or automated approaches in association with complex statistical analyses reported an improvement in differentiating HGG from SBM [24, 25]. While accurate, these algorithms may require significantly more time, experience, a dedicated workstation, and user-time.

This study has some limitations. First, it is a retrospective study that enrolled a limited sample size due to the highly specific inclusion/exclusion criteria, which produced highly homogeneous groups; i.e., groups matched for lesion characteristics and to exclude confounding parameters. We selected these stringent inclusion/exclusion criteria to have a dataset, which would be as homogeneous as possible and where differentiating between the two lesions would be a real diagnostic challenge for the radiologist.

Based on our clinical experience, we reasoned it would make less sense including both sub-centimetric SBMs and huge or midline-crossing HGG, which would not pose a real diagnostic challenge to the experienced radiologist in a real clinical setting and could “artificially” increase the accuracy of the qualitative analyses.

Second, as previously mentioned, spectroscopic and diffusion tensor imaging data were not available. The inclusion of these metrics might have further improved test performance. Third, the GM Index should be validated in a multiple center study with a larger study population. Fourth, ROI segmentation was performed manually. But a semi-automated/automated algorithm could improve accuracy while reducing the time required for diagnosis. Fifth, other more advanced approaches such as peritumoral texture analysis [28], possibly on diffusion tensor imaging metrics [29], may prove more accurate in large multicentric cohorts.

## Conclusion

In the present study, an analytically structured qualitative MRI algorithm outperformed an unstructured qualitative evaluation, for the differentiation between HGG and SBM. When using multiparametric MRI, no significant improvement was yielded when a conventional semi-quantitative evaluation was employed. These results suggest a substantial benefit of an expert analytical approach on the routine diagnostic workflow. Furthermore, the employment of the proposed data-driven quantitative algorithm allowed for superior differentiation accuracy between SBM and HGG.

## Abbreviations

HGG: High-grade glioma
SBM: Solitary brain metastasis
rCBV: Relative cerebral blood volume
Mean T2nrCBV: Mean normalized relative CBV in the peritumoral region
Mean T2PRC: Mean CBV percentage recovery in the peritumoral region
T2aPRC, T2bPRC, T2cPRC: Mean CBV percentage recovery the three ROIs in the peritumoral region
DFA: Discriminant function analysis
